# IL-12 plus CTB in intranasal DNA-MVA schemes improved magnitude and quality of both systemic and mucosal HIV cellular immune responses

**DOI:** 10.1186/1742-4690-9-S2-P5

**Published:** 2012-09-13

**Authors:** AM Rodriguez, C Maeto, J Falivene, MP Holgado, MM Gherardi

**Affiliations:** 1 INBIRS (ex Centro Nacional de Referencia para el SIDA), Buenos Aires, Argentina

## Background

Mucosal tissues are the major route of HIV transmission. Therefore, designing immunization regimes aimed to induce mucosal immune response is needed. The aim of this study was to analyze the activity of IL-12 alone or in combination with the cholera toxin B subunit (CTB), applied in DNA-prime/MVA-boost intranasal immunizations.

## Methods

Balb/c mice were intranasally immunized with DNA expressing HIV-1 EnvB plus DNAIL-12 alone or in combination with CTB (10ug, applied at prime and booster doses). Groups receiving CTB, complete cholera toxin (CT) or non-adjuvants (control) were included. All groups received MVAEnvB as boost dose. Immune responses were evaluated 14, 30 or 53 days after immunization.

## Results

IL-12 plus CTB generated the highest response, showing a synergistic effect for both adjuvants, measure by IFN-g and IL-2 ELISPOT, in spleen (7-fold increment), in regional (cervical) lymph nodes (LN), genital LN (iliac, GLN) and, more importantly, in genital tract mucosa (GT). At memory phase, we found that in the IL-12+CTB group IFN-g and IL-2 secreting cells were two to three-fold higher in both systemic and mucosal compartments (GLNs and GT) (p=0.001).

IL-12+CTB improved several quality features of the response: i) Higher levels of T-cell polyfunctionality in spleen and GT. ii) % of specific proliferating cells was increased at 10, 30 and 53 days. iii) Enhanced in vivo citotoxicity: median 53% vs 16.4% for control group. iv) Higher T-cell avidity in spleen cells (p=0.01). v) T-cell responses with a superior breadth: cross-reactivity against different Env subtypes was superior.

## Conclusion

We demonstrated that IL-12 plus CTB generated a cooperative adjuvant effect on the cellular immune response against Env applied in DNA-MVA intranasal immunizations. The improvement observed was not only in magnitude, but also in the breadth and quality of the responses induced. These results are important due to the need to develop mucosal vaccine strategies against HIV.

